# Validation of New ELISA Technique for Detection of Aflatoxin B1 Contamination in Food Products versus HPLC and VICAM

**DOI:** 10.3390/toxins13110747

**Published:** 2021-10-21

**Authors:** Elsayed Hafez, Nourhan M. Abd El-Aziz, Amira M. G. Darwish, Mohamed G. Shehata, Amira A. Ibrahim, Asmaa M. Elframawy, Ahmed N. Badr

**Affiliations:** 1 Department of Plant Protection and Biomolecular Diagnosis, Arid Lands Cultivation Research Institute (ALCRI), City of Scientific Research and Technological Applications (SRTA-City), Alexandria 21934, Egypt; elsayed_hafez@yahoo.com (E.H.); amiranasreldeen@yahoo.com (A.A.I.); 2 Department of Food Technology, Arid Lands Cultivation Research Institute (ALCRI), City of Scientific Research and Technological Applications (SRTA-City), Alexandria 21934, Egypt; nourhanm.abdo@gmail.com (N.M.A.E.-A.); amiragdarwish@yahoo.com (A.M.G.D.); 3 Nucleic Acids Research Department, Genetic Engineering & Biotechnology Research Institute (GEBRI), City of Scientific Research and Technological Applications (SRTA-City), Alexandria 21934, Egypt; asmaameg@yahoo.com; 4 Food Toxicology and Contaminants Department, National Research Centre, Dokki, Cairo 12622, Egypt; noohbadr@gmail.com

**Keywords:** aflatoxin B1, recombinant *AflR* gene, VICAM, HPLC, I-ELISA, peanut, wheat flour, milk powder

## Abstract

Toxin-contaminated foods and beverages are a major source of illness, may cause death, and have a significant negative economic impact worldwide. Aflatoxin B1 (AFB1) is a potent toxin that may induce cancer after chronic low-level exposure. This study developed a quantitative recombinant *AflR* gene antiserum ELISA technique for aflatoxin B1 detection in contaminated food products. Aflatoxin B1 residuals from 36 food samples were analyzed with HPLC and VICAM. DNA was extracted from aflatoxin-contaminated samples and the *AflR* gene amplified using PCR. PCR products were purified and ligated into the pGEM-T vector. Recombinant plasmids were sequenced and transformed into competent *E. coli* (BL21). Molecular size and B-cell epitope prediction for the recombinant protein were assessed. The purified protein was used to induce the production of IgG antibodies in rabbits. Serum IgG was purified and labeled with alkaline phosphatase. Finally, indirect-ELISA was used to test the effectiveness of polyclonal antibodies for detection of aflatoxin B1 in food samples.

## 1. Introduction

Mycotoxins are toxic secondary metabolites produced naturally by many fungi under certain growth conditions. These toxins affect metabolic processes and cause disease and death in humans and animals [1]. Toxicological actions of mycotoxins are recognized but few of these compounds or their derivatives are identified as antibiotics, growth promoters, or other drugs. Major mycotoxins include aflatoxins, gliotoxin, citrinin, ergot alkaloids, fumonisins, ochratoxin, and patulin [2].

Humans do not produce antibodies to mycotoxins and cannot be immunized against their toxicity. Nearly 25% of food becomes inedible due to contamination with mycotoxins; aflatoxins are the most serious source of contamination [3]. In 1993, aflatoxin is classified as a class one carcinogen by the World Health Organization Cancer Research Institute. Aflatoxin causes hepatotoxicity in both humans and animals. Exposure to this toxin can lead to liver cancer and death. The chemical is a bifuran toxoid produced by strains of *Aspergillus flavus* and *Aspergillus parasiticus*. About 20 derivatives are recognized, for example, B1, B2, G1, G2, M1, and M2. Aflatoxin B1 (AFB1) is the most potent and carcinogenic [4]. Aflatoxins M1 and M2 are hydroxylated metabolites of aflatoxin B1 produced by animals and commonly exist in milk and dairy products. The toxins are not common in grains. AFB1 is detected on the surfaces of maize and peanuts.

Toxicity of and exposure to AFB1 has been extensively investigated. The toxin may result in severe disease, including carcinogenesis, mutagenesis, growth retardation, and immune suppression [5]. The aflatoxin-producing fungus, *P*. *flavus,* grows and produces aflatoxins on preharvest maize and on maize in storage [6]. Peanuts are also susceptible to *Aspergillus* infection in the field or during storage. Both maize and peanuts are rich nutrient sources for these fungi [7]. Aflatoxin-contaminated agricultural products may pose serious health risks to humans and animals and negatively affect international trade [8]. According to the Food and Drug Administration (FDA) in the USA, an acceptable aflatoxin level in food is 0 ppb.

Typically, high-performance liquid chromatography (HPLC) and liquid chromatography mass spectrometry (LC-MS) are used for quantification of aflatoxins. LC-MS/MS can detect trace levels, but some limits exist. Chemical detection is slower than spectral detection (hours vs. seconds) and tedious. Professional analysts and precise chemical instruments are required [9]. Thus, accurate, rapid, full-scale detection of AFB1 is important in assessing human health and economic impacts. Evaluating contaminated food directly for specific fungi using genes that control aflatoxin is a promising strategy [10]. Gallo et al. [11] reported such genes in the genome of aflatoxin-producing fungi. However, the authors’ method required costly instruments, amplification, isolation, and quantification along with trained personnel [12]. The method is quite complex and costly for routine use. This study aimed to develop a new cost- and time-effective quantitative method using modified recombinant *AflR* gene antiserum enzyme-linked immunoassay (ELISA) for aflatoxin B1 detection in contaminated food products.

## 2. Results and Discussion

### 2.1. The Aflatoxin B1 Detection with HPLC and VICAM

VICAM was less sensitive than HPLC analysis for aflatoxin AFB1 detection in several samples—peanut 2, flours 2 and 3, and milk-powder 3. HPLC is widely used for the analysis of aflatoxins for sensitivity and accuracy [13] (Table 1). HPLC is an excellent quantitative method in detection of aflatoxins [14], although it requires skilled operators, extensive sample preparation, and is a high-cost equipment [15].

### 2.2. Molecular Detection and SDS-PAGE

**Figure 1:**
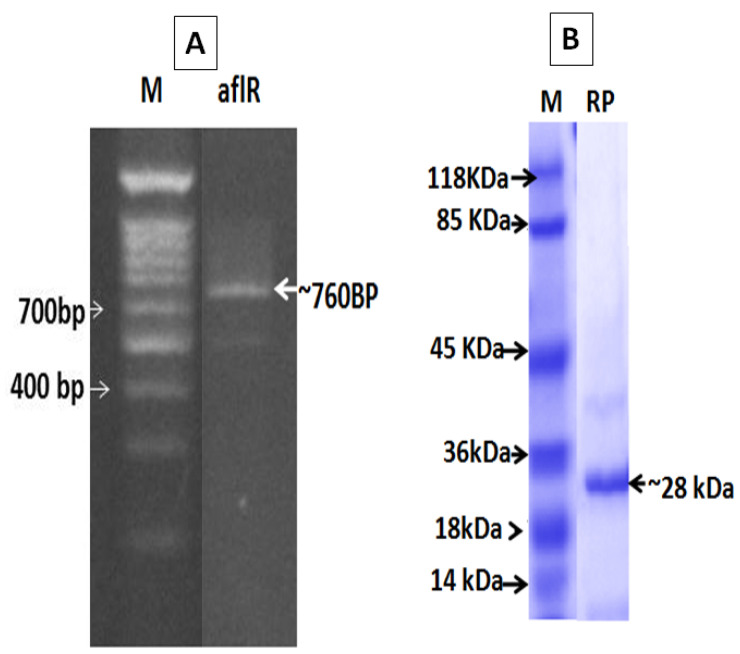
(**A**) PCR product amplified using the specific primers of the afIR gene (Aflatoxin B1). M: DNA marker and afIR the amplified gene in molecular size about 760 bp. (**B**) The recombinant protein of the in vitro transcribed afIR gene (Aflatoxin B1) with molecular size about 28 kDa.

A unique band at about 760 bp was observed in all positive samples (Figure 1A). Negative amplification was observed in flour contaminated with aflatoxin (50, 75, and 100 mg). Amplified DNA was cloned and in vitro transcribed protein was separated on SDS-PAGE (Figure 1B). SDS-PAGE analysis revealed a protein of about 28 kDa. Molecular weight determination regarded as first characterization step of protein was further used in toxin detection.

### 2.3. Sequence Analysis and Phylogenic Construction

**Figure 2:**
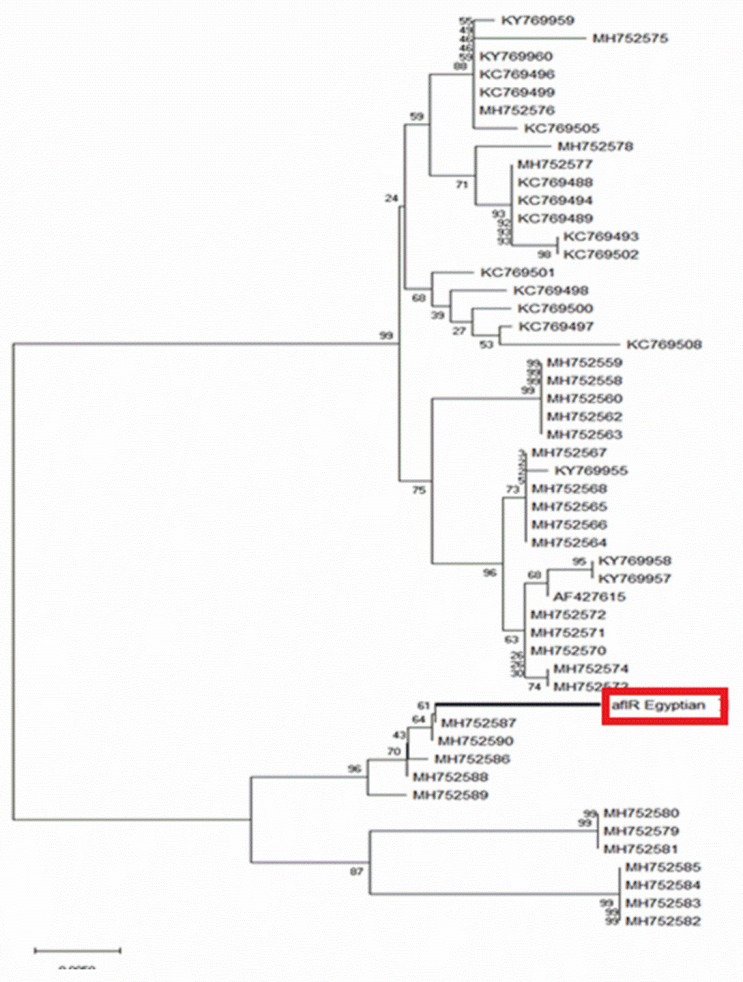
Phylogenetic tree for the amplified aflatoxin B1 based on the DNA nucleotide sequence and compared with the other 50 *AFB1*genes listed on gene bank. The phylogeny was constructed using Mega 6 program.

The PCR product was purified and sequenced and a 750 bp fragment was obtained. The sequence was aligned using the NCBI analysis tool and showed 97% similarity with other *AfIR* genes listed in GeneBank. The sequence was compared with 50 sequences and a phylogenetic tree was constructed. An Egyptian *AfIR* gene was closely similar to *AfIR* gene MH752587 obtained from *Aspergillus* sp. PS-2018c isolate BN038G *AFlR*, Arizona, USA (Figure 2).

### 2.4. Antigenicity Test

**Figure 3:**
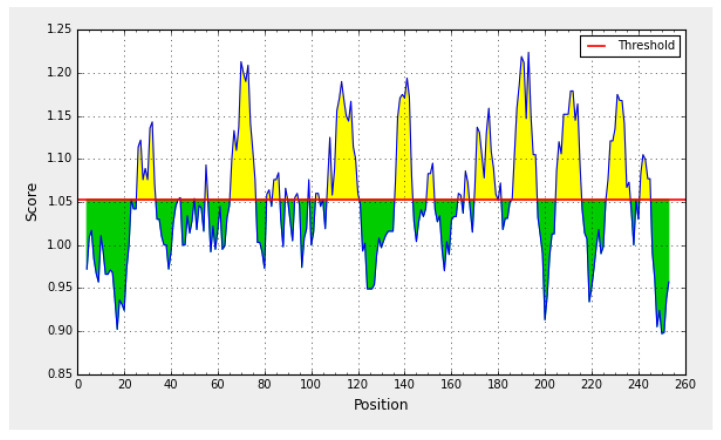
The predicted antigenic activity of the recombinant protein (afIR).

A 256 residue amino acid sequence was deduced from the gene sequence (Table 2). Eight peptides showed antigenic activity by Kolaskar & Tongaonkar Antigenicity [16] Figure 3. Peptide lengths ranged from 8 to 14 amino acids. Their positions started from amino acid numbers 26, 66, 107, 136, 170, 186, 205, and 236 (Table 2). Epitope prediction using Kolaskar and Tongaonkar Antigenicity Prediction identifies the protein epitopes that are useful for diagnostic purposes and also in the development of peptide vaccines [17].

### 2.5. IgG Polyclonal Antibody Labeling and Purification

Serum obtained from immunized rabbits was fractionated using affinity chromatography protein G-Sepharose column and one band of conventional IgG with a molecular weight of 130 kDa was obtained. Moreover, two bands of a 42 kDa heavy chain and a 19 kDa light chain were separated under reducing conditions. Glutaraldehyde was used to prepare conjugates using a ratio of 4:1 of IgG and enzyme alkaline phosphatase (AP). IgG-AP conjugates were purified by gel filtration on a Sephacryl S200 column. AP (EC 3.1.3.1) is a stable enzyme its activity can be measured by many different substrates. The most common method of labeling immunoglobulin G (IgG) antibody with this enzyme uses the homobifunctional reagent glutaraldehyde [18].

### 2.6. Validation of the Modified Recombinant AflR Gene Antiserum ELISA Technique with HPLC and VICAM

In definition, validation is establishing the performance specifications of a new diagnostic tool such as a new test, laboratory developed test or modified method. But verification is defined as one-time process to determine performance characteristics of a test before use in patient testing [19].

ELISA was unable to distinguish among antigens due to the presence of common epitopes on protein surfaces [20,21,22]. Sampling/sub-sampling variation significantly affects the accuracy of aflatoxin analysis [23]. Extracts of 36 samples were used for validation to minimize the impacts of such variation.

**Figure 4:**
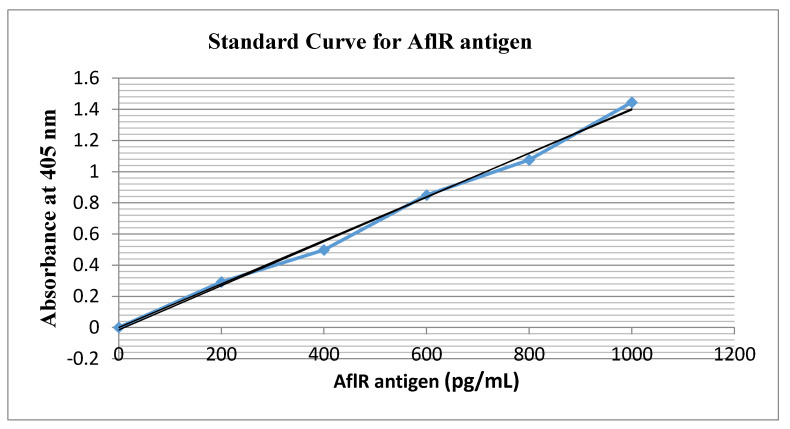
I-ELISA standard curve for *AflR* recombinant protein using purified serum IgG.

Recombinant antiserum detected *AfIR* recombinant protein within a concentration range 0–1000 pg/mL with a linear correlation between *AfIR* antigenic protein and absorbance at 405 nm (y = 0.0014x − 0.0148; R^2^ = 0.9946) (Figure 4). Non-significant differences among three samples of the same product, peanut, flour, or milk powder, were observed after HPLC (*p* > 0.05) (Table 3). The VICAM method showed similar results. However, the modified ELISA showed significant differences among toxin detections in these product samples. The serum-based analysis confirmed specific PCR results. No false negatives were observed in I-ELISA results and false positives were either nil or negligible.

**Figure 5:**
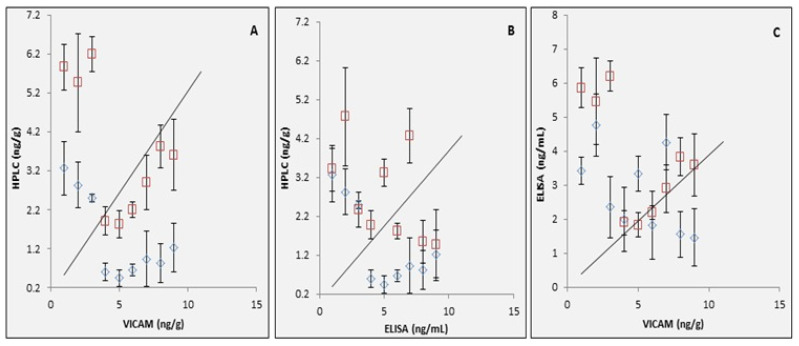
Correlations between HPLC and VICAM (**A**), HPLC and ELISA (**B**), ELISA and VICAM (**C**).

Although the correlation between the data in Figure 5A,B (comparing HPLC against VICAM and ELISA) reflected that the correlation of HPLC against VICAM (Figure 5A) was better than ELISA. On the other hand, a good correlation was observed between ELISA and VICAM (Figure 5C). However, the represented modified ELISA technique is easier to use, economic as it does not need sophisticated chemicals or highly trained technicians, have a good sensitivity to detect low infection levels determining aflatoxin B1 in foods and can represent a successful alternative in case other approaches are hard to be reached in less developed communities. Previous observations were reported for validation of a competitive direct SUNQuik ELISA for aflatoxin in peanuts using a reference HPLC method and other methods, including a minicolumn and the VICAM Afla test system [24]. The comparison between HPLC, VICAM, and validated method I-ELISA with respect to limit of detection, precision and accuracy, time of analysis, cost of analysis, and use of organic solvents is summarized in Table 4.

### 2.7. Limitations of the Modified Recombinant AflR Gene Antiserum ELISA Technique

Our new established method has many limitations that must be clarified to determine and specify the application field of this method. First, our new ELISA technique does not measure the aflatoxins itself, hence, this type of test cannot be used for official control. However, it could be useful for auto control and rapid results and decision-making within a farm/company. Second, although this method is quantitative test, all positive results need to be confirmed with a confirmatory method such as HPLC or LC-MS as it is based on the measurement of a recombinant protein controlled by the gene responsible for aflatoxin biosynthesis, but not on the toxin itself.

## 3. Conclusions and Future Perspective

Aflatoxin B1 detection is an increasingly important health and economic issue. Accurate detection is essential to assess health problems in both humans and animals. Conventional detection methods are time-consuming and require expensive chemicals and apparatus (HPLC and VICAM). An accurate and rapid detection method that requires fewer chemicals is needed. We developed a specific quantitative detection technique (I-ELISA) using recombinant *AflR* protein. *AflR* is involved in aflatoxin biosynthesis. Comparison of results achieved from the new modified ELISA with other standardized methods HPLC and VICAM, revealed that the new ELISA technique can be used at many applications as an economic alternative to detect low levels of aflatoxin contamination. This modified technique may address problems associated with the reliable and rapid detection of aflatoxin B1 contamination in food products. The technique could be used to develop highly sensitive (0–1000 pg/mL) testing capabilities. In future, hybridoma cell culture antibody production technique can be used for production of antibodies against AfIR protein for large-scale manufacturing of rapid I-ELISA kit. This method will yield a production scale ranging from milligram to gram level with competitive pricing.

## 4. Materials and Methods

### 4.1. Sampling

Thirty-six food samples of three food products (12 samples each) were collected from a local market in Alexandria, Egypt. Products were prepared by different companies (4 packages each). Samples were peanuts (300 g packages), wheat flour (2 kg packages), and milk powder (500 g packages). Aflatoxin B1 was extracted for subsequent analysis.

### 4.2. HPLC Detection

One mL of each sample was centrifuged at 6000 rpm for 15 min, then filtered through a 0.45 μm hydrophobic polytetrafluoroethylene syringe filter in preparation for gel pores chromatographic (GPC) analysis. The supernatant was transferred to 1.5 mL micro-tubes and passed through an immune-affinity column at a rate of 1–2 drops/s. The column was washed twice with 10 mL water: methanol (90:10) at a flow rate of 3 mL/min. Aflatoxins were eluted by slowly passing 1 mL of methanol through the column. The clear eluent was then repassed through a 0.45 μm filter [25]. Subsequently, 100 μL of trifluoracetic acid and 200 μL n-hexane were added to samples and mixed by vortexing for 30 s. The vial was left for 15 min before addition of 900 μL of water: acetonitrile, 9:1 and remixing by vortexing. The hexane layer was then removed and samples were analyzed for AFs as previously reported [26] using a Waters HPLC system, Model 6000, a solvent delivery system, and a Model 720 system controller equipped with a fluorescence detector (Model 274) at excitation and emission wavelengths of 360 and 450 nm, respectively. Separation used 5 μm of sample, a Waters symmetry column (150 × 4.6 mm id), and a flow rate of 1 mL/min with an isocratic system of 1% acetic acid: methanol: acetonitrile (55:35:10).

### 4.3. AflA-Vt Detection

Afla-V strip tests utilize the proven sensitivity and selectivity of VICAM monoclonal antibodies to accurately detect and measure aflatoxins B1 at levels of 2 to 100 ppb. These samples were subjected to aflatoxin extraction and quantification using the VICAM fluorometry method. Briefly, representative samples (100 g) of shelled peanuts were added with 10 g of NaCl and 200 mL of methanol/water (80:20 *v*/*v*), homogenized using a Waring blender at high speed for 1 min and filtered through Whatman paper. Five ml of the filtrate was diluted with 20 mL HPLC water then re-filtered. Ten milliliter filtrate was purified with VICAM immunoaffinity columns (VICAM Aflatest, MA, USA) containing aflatoxin-specific (B1) monoclonal antibodies and washed with 10 mL HPLC water before the aflatoxin was eluted with 1 mL methanol. The eluted fraction was diluted twice with HPLC water and measured with the VICAM fluorometer (VICAM Series 4EX Fluorometer). All procedures were done according to the manufacturer’s instructions [27].

### 4.4. Specific PCR Detection Method

DNA from food samples was extracted using a QiaGene DNA extraction kit (QiaGene, Berlin, Germany). DNA was dissolved in DEPC-treated water, quantified spectrophotometrically and analyzed using 1.2% agarose gels. The AflR gene (744 bp) was amplified using specific primers. The PCR reaction consisted of 1 µL of DNA in 2.5 µL Taq polymerase buffer 10× (Promega, New York, NY, USA) containing a final concentration of 1 mM MgCl_2_, 0.2 Mm dNTPs, 20 pmol of each primer, and 0.2 µL Taq polymerase (5 U/µL) in a final reaction volume of 25 µL. The PCR reaction program was: initial denaturation at 95 °C for two minutes followed by 35 cycles of 58 °C for one min, 72 °C for one min, and 95 °C for 2 min. A final extension step at 72 °C for 5 min was included at the end of the reactions. PCR amplification products were separated in 1.5% agarose with 0.5× TBE buffer and visually analyzed with a gel documentation system (Syngene) [28]. Forward (5‘-TAAGCAGAATTCGAATAGCTTCGCAGGGTGGT-’3) and reverse (5‘-GAATAGCTTCGCAGGGTGGTGCGGCCGCTAAGCA-’3) primers were designed by Primer-Blast, NCBI.

### 4.5. Detection via AflR Gene Analysis and Transformation

#### 4.5.1. Cloning, Sequencing, and *AflR* Gene Transformation

The PCR product (Section 2.4) was excised from the gel and purified using a QIA quick gel extraction kit (Qiagen Inc., Berlin, Germany). Purified DNA was ligated into the pGEM-T vector (Promega Co., New York, NY, USA). Recombinant plasmids were directly sequenced using an automated sequencer (Macrogene Company, Seoul, Korea), with a universal vector primer. DNA homology searches were carried out using the NCB1 databases and the BLAST network service. *EcoR*I and *No*tI restriction enzymes were used for gene release and insertion into the pPROEX HTa expression vector (Life Technologies, New York, NY, USA). The recombinant plasmid was transformed into competent *E. coli* (BL21) cells and recombinant protein was recovered as previously described [29].

#### 4.5.2. Molecular Size Determination of *AflR* Recombinant Protein

The recombinant protein was separated on 12% SDS PAGE and molecular size determined using a standard low range protein marker (BioRad, Hercules, CA, USA). Gel preparation, staining, and destaining were carried out following Laemmli [30].

#### 4.5.3. Epitope Prediction and Antigenic Determination

B-cell epitope prediction analysis was performed following Kolaskar and Tongaonkar [16] to examine the epitope in different antigenic determinants.

#### 4.5.4. Immunization and Antibody Production

##### Rabbit Immunization with *AflR* Recombinant Protein

**Figure 6:**
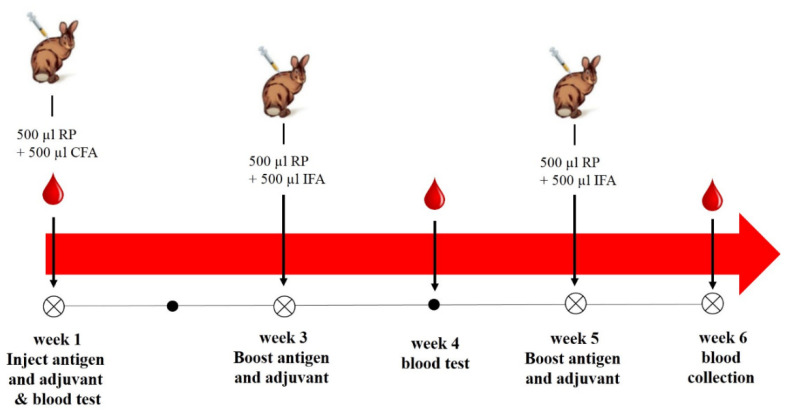
Polyclonal antibody production protocol.

Ten male New Zealand White rabbits, age 10–16 weeks and weighing 3.5–4.0 kg were used. Physical examinations confirmed lack of abnormalities. Rabbits were housed in stainless steel and polycarbonate cages (Techniplastic, West Chester, PA, USA), at 18–21 °C, with 30–70% humidity, and a 12-h: 12-h light: dark cycle (lights on at 0600). Rabbits were fed 250 g of a commercial pelleted rabbit diet (diet 2030, Harlan Laboratories, Madison, WI, USA) twice daily and were allowed free access to municipal water via an automatic watering system (Edstrom Industries, Waterford, WI, USA). After one-week of acclimatization, rabbits were divided into control (4 animals) and treated (6 animals) groups. The latter animals were injected subcutaneously with 500 µL of purified protein (2 mg/mL) following the polyclonal antibody production protocol of Fishback et al. [31] with some modifications (Figure 6). The study was conducted after obtaining approval from the International Animal Care and Use Committees (IACUCs) IACUC # 30-1Y-0521 (date of approval 10 January 2018).

Three milliliter of blood was collected from the auricular artery of each rabbit on weeks 1 and 4 to monitor antibody production. On week 6, under deep anesthesia with a mixture of 22–50 mg/kg ketamine and 5–10 mg/kg xylazine, 3 mL of blood was collected by cardiac puncture. Blood was collected into BD Vacutainer serum separation tubes (BD, Franklin Lakes, NJ, USA), and kept upright at room temperature (20 ± 2 °C) for serum separation following the manufacturer’s instructions. Separated sera were stored at −80 °C until further analysis.

##### Serum IgG Purification and Fractionation

Rabbit sera were obtained by centrifugation of immunized rabbit blood at 4000 rpm for 5 min at 4 °C. IgG fractions were obtained by loading sera onto an affinity Protein G-Sepharose column. In brief, the IgG1 fraction was eluted with glycine buffer, pH 2.7, and the IgG3 fraction obtained by elution with glycine buffer, pH 3.5. All IgG fractions were immediately neutralized in a neutralization buffer (1 M Tris–HCl, pH 8.0, 150 mM NaCl, 5 mM EDTA) [32].

##### Labelling of Antibodies

Ten mg of alkaline phosphatase (AP) were mixed with purified IgGs (2.5 mg) in 5 mL of 50 mM phosphate buffer, pH 6.8. Mixtures were dialyzed against 2 L of 50 mM phosphate buffer for 24 h at 4 °C. One mL of 1.25% glutaraldehyde was added to each mixture and gently stirred for 2 h at room temperature (20 °C ± 2). Two hundred fifty µL of 0.2 M glycine solution was added followed by further stirring for 2 h. Mixtures then were dialyzed twice against 2 L of 1.0× PBS containing 1 mM magnesium chloride, followed by centrifugation at 10,000 rpm for 5 min to remove any precipitate [33]. Each conjugate was further purified on a Sephacryl S200 column (5 × 150 mm, GE Health care, Danderyd, Sweden) previously equilibrated with PBS and eluted with the same buffer.

#### 4.5.5. Quality Checks

**Figure 7:**
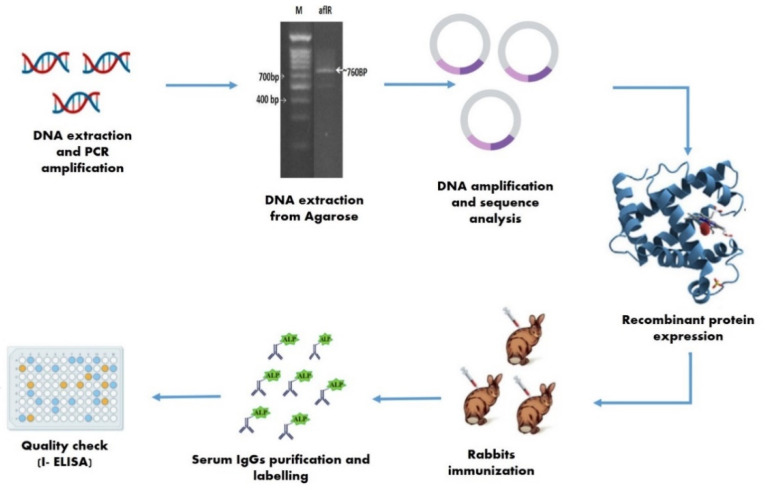
Summary of experimental steps.

An indirect enzyme-linked immunosorbent assay (I-ELISA) was used to detect aflatoxin B1 in food samples using polyclonal antibodies. Antibodies were compared using an antiserum produced by Sigma (Berlin, Germany). One gram of contaminated food sample was extracted in 10 mL coating buffer. One hundred microliter of sample extract was added to each well. Plates were then incubated at 37 °C for 3 h and blocked with 200 μL of blocking buffer (1× PBS and 0.5% BSA) for 1 h at room temperature (20 °C ± 2). One hundred microlite concentration of 1:800 diluted secondary antibody alkaline phosphatase-conjugated (anti-rabbit antibody) was added and the mixture was incubated at 37 °C for 1 h. All washing steps between incubations used 1× PBS-T buffer. Finally, freshly prepared pNPP substrate was added; plates were incubated at room temperature for 30 min away from direct light, and the absorbance was measured at 405 nm. All experimental steps are summarized in Figure 7.

## 5. Statistical Analysis

Data were statistically analyzed using SPSS software (version 16). One-way analysis of variance was used to assess the significance of differences among means, with a significance threshold of *p* < 0.05. The Pearson correlation coefficient (r) was also calculated (*p* < 0.001) to assess the strength of linear relationships between variables.

## Figures and Tables

**Table 1:** HPLC and VICAM screening analysis of 36 food samples for aflatoxin detection.

Samples	HPLC	VICAM
Peanut 1	+++	+++
Peanut 2	+++	++
Peanut 3	+++	+++
Flour 1	++	++
Flour 2	+++	++
Flour 3	++	+
Milk-powder 1	++	++
Milk-powder 2	+	+
Milk-powder 3	++	+

+: refers to low aflatoxin contamination level; ++: refers to moderate aflatoxin contamination level; +++: refers to high aflatoxin contamination level.

**Table 2:** Predicted peptides with antigenic activity, their length, and positions.

No.	Start	End	Peptide	Length	>aflIR d Deduced Amino Acid Sequence
1	26	33	LMQVPKIY	8	MSHSYNTFAGWFINTPTGRTQGSLALMQVPKI
2	66	76	EHYLLFLVQFV	11	YLAGNKSFLGSQPAHDGLRYLEPEACMRAGQL
3	107	120	TPQLVTFVYIHLDL	14	AEHYLLFLVQFVNRSRSSLVTRFQPRYVNKEC
4	136	143	FTLCVPPRLA	8	TARQSLGQVRTPQLVTFVYIHLDLSARQRKGO
5	170	179	PGRCVPPPRLA	10	ATLQEKAFTLCVFFPAFNSKLYSTPSSRPPRW
6	186	196	IAVRVVPVQKC	11	LTIFPPGHIPGRCVPPRLAALESSGIAVRVVPVQKC
7	205	215	VLGVSNVVLPV	11	DAPRRNRPVLGVSNVVLPVNTWSPSGWAAT
8	227	236	RALPVPLIQL	10	RALPVPLIQLGDHQRVFLQPDRNRDIRRIT

**Table 3:** Comparative results obtained by HPLC, VICAM, specific PCR, and I-ELISA (ng/g).

Sample	HPLC (ng/g)	VICAM (ng/g)	Specific PCR	ELISA (ng/mL)
Peanut 1	3.26 ± 0.68 ^a^	5.86 ± 0.58 ^a^	+	3.43 ± 0.40 ^a,b^
Peanut 2	2.83 ± 0.58 ^a^	5.46 ± 1.26 ^a^	+	4.76 ± 0.92 ^a^
Peanut 3	2.50 ± 0.10 ^a^	6.20 ± 0.45 ^a^	+	2.36 ± 0.90 ^b,c^
Flour 1	0.60 ± 0.23 ^b^	1.90 ± 0.36 ^c^	+	1.98 ± 0.94 ^b,c^
Flour 2	0.44 ± 0.22 ^b^	1.83 ± 0.35 ^c^	+	3.33 ± 0.51 ^a,b^
Flour 3	0.66 ± 0.15 ^b^	2.20 ± 0.20 ^c^	+	1.82 ± 1.01 ^c^
Milk-powder 1	0.93 ± 0.71 ^b^	2.90 ± 0.70 ^b,c^	+	4.26 ± 0.81 ^a^
Milk-powder 2	0.82 ± 0.50 ^b^	3.83 ± 0.55 ^b^	+	1.55 ± 0.67 ^c^
Milk-powder 3	1.23 ± 0.62 ^b^	3.60 ± 0.91 ^b^	+	1.46 ± 0.84 ^c^

The mean values indicated in the same column within variable with different superscripts (a, b, and c) were significantly different (*p* < 0.05); +: present of fungal infection.

**Table 4:** Comparison between HPLC, VICAM, and validated method I-ELISA.

Parameters	HPLC	VICAM	I-ELISA
Limit of detection	<0.008 ng/mL	<2 ng/mL	<1 ng/mL
Time of analysis	120 min	90 min	30 min
Cost of analysis	High	Moderate	Moderate
Use of organic solvents	Yes	Yes	No
